# Chromogranin A as a Marker for Diagnosis, Treatment, and Survival in Patients With Gastroenteropancreatic Neuroendocrine Neoplasm

**DOI:** 10.1097/MD.0000000000000247

**Published:** 2014-12-12

**Authors:** Yu-hong Wang, Qiu-chen Yang, Yuan Lin, Ling Xue, Min-hu Chen, Jie Chen

**Affiliations:** From the Department of Gastroenterology, The First Affiliated Hospital of Sun Yat-Sen University, 58 Zhongshan II Road, Guangzhou, China (YHW, QCY, MHC, JC); and Department of Pathology, the First Affiliated Hospital of Sun Yat-sen University, 58 Zhongshan II Road, Guangzhou, China (YL, LX).

## Abstract

Chromogranin A (CgA) not only plays an important role in pathologic diagnosis, but is also used as a circulating biomarker in patients with gastroenteropancreatic neuroendocrine neoplasm (GEP-NEN). However, the relationship between immunohistochemistry (IHC) expression and serum levels of CgA has not been investigated. The value of CgA for evaluating treatment response and prognosis is still not well understood. We conducted this study to assess the significance of CgA in GEP-NEN in terms of diagnosis, curative effects evaluation and prognosis.

One hundred forty-five patients comprising 88 patients with active disease and 57 disease-free patients were enrolled in this study from January 2011 to November 2013. The expression of CgA was assessed by IHC, and serial serum CgA levels were measured by enzyme linked immunosorbent assay.

The overall expression rate of CgA was 69.0% (100/145). CgA expression was associated with tumor site and stage (*P* < 0.05), but not correlated with prognosis (*P* = 0.07). Serum CgA levels were significantly higher in GEP-NEN patients with active disease when compared with disease-free patients (*P* = 0.001) or healthy participants (*P* < 0.001). A CgA cutoff value of 95 ng/ml discriminated between healthy subjects or disease-free patients and patients with active disease (sensitivity 51.2% and specificity 87.5%, respectively). There was a correlation between the CgA IHC expression and high serum CgA levels (*R* = 0.320, *P* = 0.002). Serum CgA levels were much higher in patients who classified as neuroendocrine carcinoma, mixed adenoendocrine carcinoma (*P* = 0.035) and who were on stage IV (*P* = 0.041). Changes in CgA levels normalization or ≥30% decrease suggested that patients had tumor response. Furthermore, patients with serum CgA levels higher than 95 ng/ml had a significantly shorter survival compared with patients with levels lower than 95 ng/ml (*P* < 0.001).

CgA is a reliable pathologic and circulating maker for diagnosis of GEP-NEN. We further confirmed that serial measurement of CgA may be useful for evaluating the efficacy of different kinds of therapies in patients during follow-up, and serum CgA level ≥95 ng/ml may serve as a predictor of overall survial.

## INTRODUCTION

Gastroenteropancreatic neuroendocrine neoplasms (GEP-NENs) have a wide spectrum of clinical presentations, which range from clinically silence to tumor-producing peptide-related symptoms such as flushing or diarrhea. Although they are generally more indolent than carcinomas, they are often associated with a very aggressive clinical course and 60% to 80% of NENs are metastatic when identified.^1^ Therefore, to obviate delay and ensure early recognition, their early diagnosis requires attention.

Chromogranin A (CgA) is a 49-kDa acidic glycoprotein that belongs to the granin family, a principal component of dense-core granules in neuroendocrine cells. Its expression generally correlates with the number of dense-core granules in neuroendocrine cells. CgA and hormones are co-secreted from neuroendocrine cells during the secretory granule exocytotic process. CgA itself can also be degraded into a series of smaller biologically active peptides, such as pancreastatin, catestatin, and vasostatins I and II.^1^ Recently, the newest NEN classification systems such as World Health Organization (WHO) 2010 classification, European Neuroendocrine Tumor Society (ENETS) and the North American Neuroendocrine Tumor Society report that immunohistochemical (IHC) detection of CgA should be performed to confirm the ‘neuroendocrine’ character of tumor cells.^2–4^ CgA is also used as a circulating marker. Previous studies have shown that elevated circulating CgA levels were demonstrated in serum or plasma of patients with various neuroendocrine tumors. The sensitivity and specificity of elevated CgA for the diagnosis of GEP-NEN range from 60% to 100%.^5–8^ It has also been suggested that CgA may be a precious tool for predicting recurrences and monitoring the follow-up.^1,6,9^

However, the relationship between IHC expression and serum levels of CgA has not been investigated. In addition, the value of serial determinations of CgA for evaluating treatment response in patients with different therapies is still not well understood. Furthermore, previous studies looking at the prognostic capability of CgA have shown conflicting results.^10–12^ In this study, we aimed to evaluate the significance of CgA in patients with GEP-NEN in terms of diagnosis, therapeutic response evaluation and prognosis, and assess the relation between the expression and high serum levels of CgA.

## PATIENTS AND METHODS

### Patient Information

A total of 145 patients with histologically confirmed sporadic GEP-NEN in The First Affiliated Hospital, Sun Yat-sen University from September 2002 to November 2013 were enrolled to analyze CgA expression and serum CgA levels. The 145 patients (87 men and 58 women) had a median age of (51 ± 14) years (range 18–85). The most common primary tumor site was the pancreas (41/145, 28.3%). Gastrointestinal NENs accounted for 63.4% of GEP-NEN. The remaining sites included metastatic NENs of unknown primary, retroperitoneum, gallbladder, etc. (8.3%). Seventeen patients (11.7%) had functioning tumors, of which insulinoma comprised 47.1%. Among all the cases, 56 patients (40.6%) had G1 tumors, 41 (29.7%) had G2 tumors, and 41 (29.7%) had G3 tumors, respectively. The remainder seven patients were unable to be graded because of small needle biopsy samples. Neuroendocrine tumor (NET), neuroendocrine carcinoma (NEC), and mixed adenoendocrine carcinoma (MANEC) were 70.3%, 29.0%, and 0.7%, respectively. Regarding the TNM staging, 44 patients (30.3%) were on stage I, 19 (13.1%) on stage II, and 21 (14.5%) on stage III. Sixty-one patients (42.1%) who had distant metastases were on stage IV.

A functional tumor was defined as a tumor-overproducing hormone that causes clinical symptoms. The pathology of each patient was reviewed according to the latest WHO classification of tumors of digestive system.^2^ TNM stage was adopted according to the ENETS Consensus Guidelines^13,14^ (Tumors which did not include in the ENETS TNM stage system were classified by 2012 American Joint Committee on Cancer Staging Atlas^15^). Curative surgery was defined as complete resection of the primary tumor with clear margins. Systemic therapies comprised cytotoxic chemotherapy, somatostatin analog, and targeted agent. Treatment responses were evaluated using imaging studies according to RECIST 1.1 criteria.^16^

### Immunohistochemistry

CgA IHC stains were performed in all 145 cases. Sections (4 μm) of formalin-fixed paraffin-embedded tumor specimens were deparaffinized in xylene and rehydrated in graded alcohol. Endogenous peroxidase activity was blocked by incubating the slides in 3% hydrogen peroxide for 20 min at room temperature. The slides were then rinsed under running water for 5 min. Heat-induced epitope retrieval was carried out using a microwave oven at 199°F for 30 min in preheated 10 mmol/L citrate buffer (pH 6.0). The slides were transferred to phosphate-buffered saline and then incubated at 4°C with rabbit polyclonal antibodies against CgA (1:50; DaKo) overnight. Next day, the samples were incubated in secondary antibody for 1 h at room temperature. The substrate chromogen, 3.3°-diaminobenzidine, enabled visualization of the complex via a brown precipitate. Hematoxylin (blue) counterstaining enabled the visualization of the cell nuclei. Omission of primary antibody served as a negative control. All slides were evaluated independently by two investigators (YL and LX) who were blinded to the patients’ clinical data.

### Serum CgA Determination

All patients were divided into active disease (patients with advanced or recurrent disease, n = 88) and disease-free (patients without residual or recurrent disease after surgery, n = 57) groups after analyzing the serum CgA levels in GEP-NEN. Serial CgA levels and imaging studies were performed every 2 to 3 months for 45 patients who received curative surgery or systemic therapy. Eighty-four healthy subjects, without evidence of NEN, malignancies, hypertension, renal, or liver failure, and not treated with proton pump inhibitors at the time of CgA measurement, were enrolled in the study. The 84 healthy subjects (40 men and 44 women) had a median age of (51 ± 11) years (range 21–83). No difference was observed in terms of sex or age between GEP-NEN patients and healthy subjects.

Blood samples were obtained in the early morning after an overnight fast and collected before treatment for patients who received curative surgery or systemic therapy. All blood were centrifuged at 3000 g within 30 min. Serum was frozen in aliquots and kept at −20°C before analysis.

Measurement of serum CgA levels was performed with a commercial kit (chromoa assay; CIS Bio International, Saclay, France).The chromoa assay is based on a sandwich enzyme linked immunosorbent assay, and it uses two monoclonal antibodies directed against the CgA amino acid sequences 145 to 197 and 198 to 245.

### Statistical Analysis

The association of CgA expression with various clinicopathologic features was analyzed using Pearson chi-square test. To investigate the diagnostic value of serum CgA, receiver operating characteristic (ROC) curves were plotted, and the area under the curve (AUC) was calculated. The correlation between CgA expression and serum levels was carried out by Spearman rank order correlation. Serum CgA levels between subgroups, which are presented as median, were compared using the Mann–Whitney or Kruskal–Wallis test. Survival analysis was performed using Kaplan–Meier survival plots and comparisons between groups were made with the log-rank test. CgA expression was analyzed in two groups, positive and negative controls; while serum CgA was analyzed in the two groups above or below the diagnostic cutoff value. Statistical analysis was performed using SPSS 16.0 software (SPSS Inc.). *P* < 0.05 was considered statistically significant.

### Ethics

The study was approved by the ethics committee of The First Affiliated Hospital Sun Yat-sen University (with a reference number: [2012]317) and complied with the Declaration of Helsinki. Written informed consent was obtained from the patients.

## RESULTS

### CgA Expression in GEP-NEN

**FIGURE 1:**
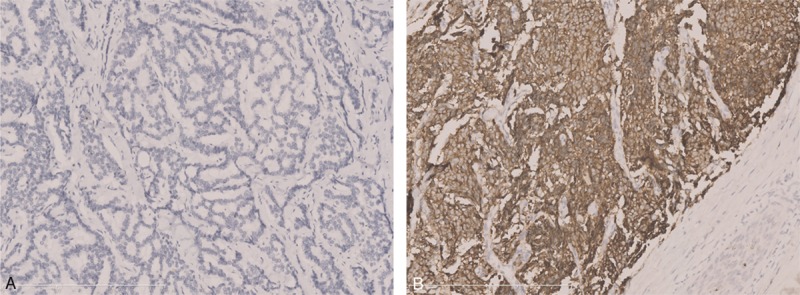
Immunohistochemical staining of CgA. (A) No expression of CgA in rectal NEN and (B) high expression of CgA in pancreatic NEN.

As shown in Figure 1, CgA was positively immunostained in the cytoplasm of tumor cells. The overall expression rate of CgA was 69.0% (100/145). CgA expression was probably higher in pancreatic neuroendocrine neoplasms than in gastrointestinal neuroendocrine neoplasms (*P* = 0.05). The expression rate of CgA in tumors on stage II was 84.2%, which was much higher than tumors on stage IV (77.0%), stage I (59.1%), and stage III (52.4%) (*P* = 0.035). No other correlations of the expression of CgA with clinicopathologic variables in GEP-NEN were evident (Table 1).

**TABLE 1:**
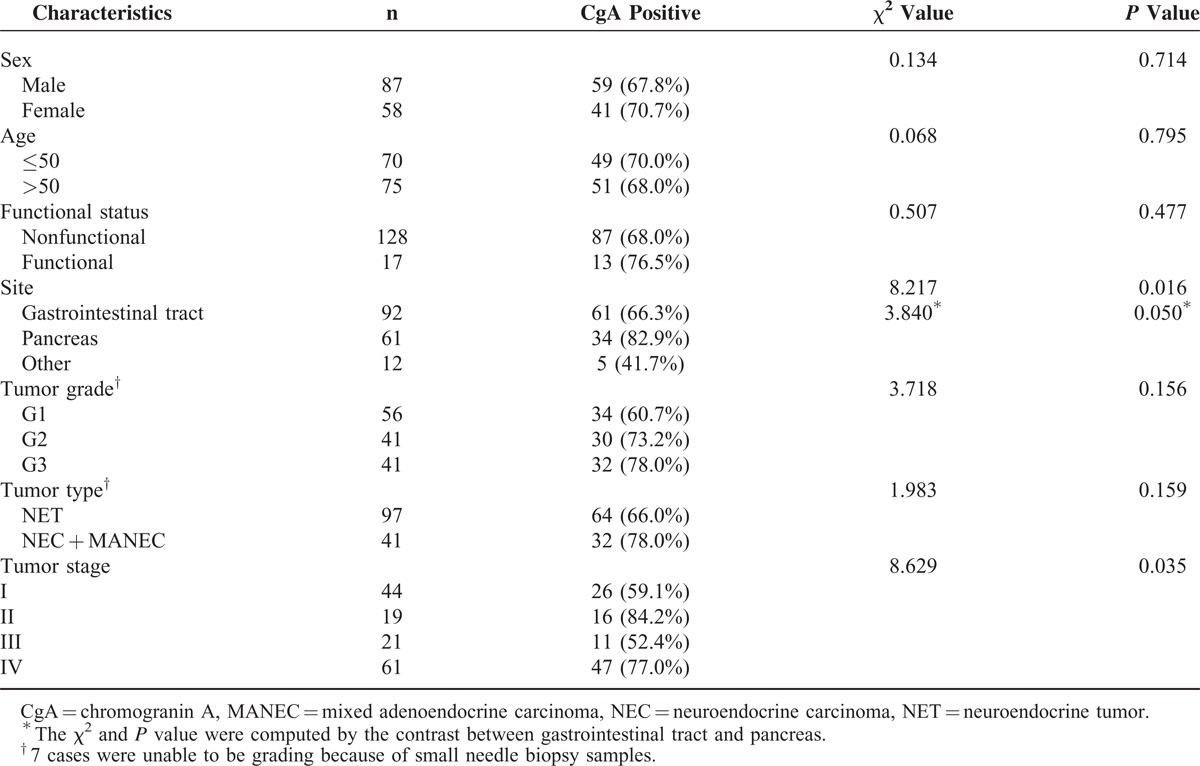
Chromogranin A Expression in Gastroenteropancreatic Neuroendocrine Neoplasm and Correlation With Clinicopathologic Variables (N = 145)

### Serum CgA Levels in GEP-NEN Patients and Healthy Subjects

**FIGURE 2:**
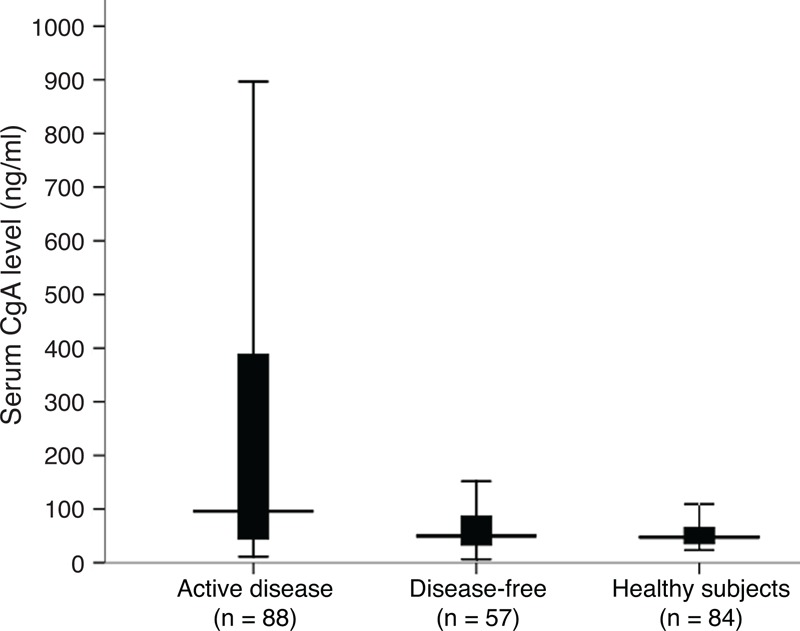
Serum CgA levels in active disease, disease-free, and healthy subjects. CgA = chromogranin A.

Serum CgA levels in active disease, disease-free, and healthy subjects are shown in Figure 2. The median serum CgA level of patients with active disease was 96 ng/ml, which significantly higher than of disease-free patients (50 ng/ml, *Z* = 3.242, *P* = 0.001) or healthy subjects (48 ng/ml, *Z* = 4.002, *P* < 0.001). No difference in CgA levels was evident between disease-free patients and healthy subjects.

### Diagnostic Property of CgA

**FIGURE 3:**
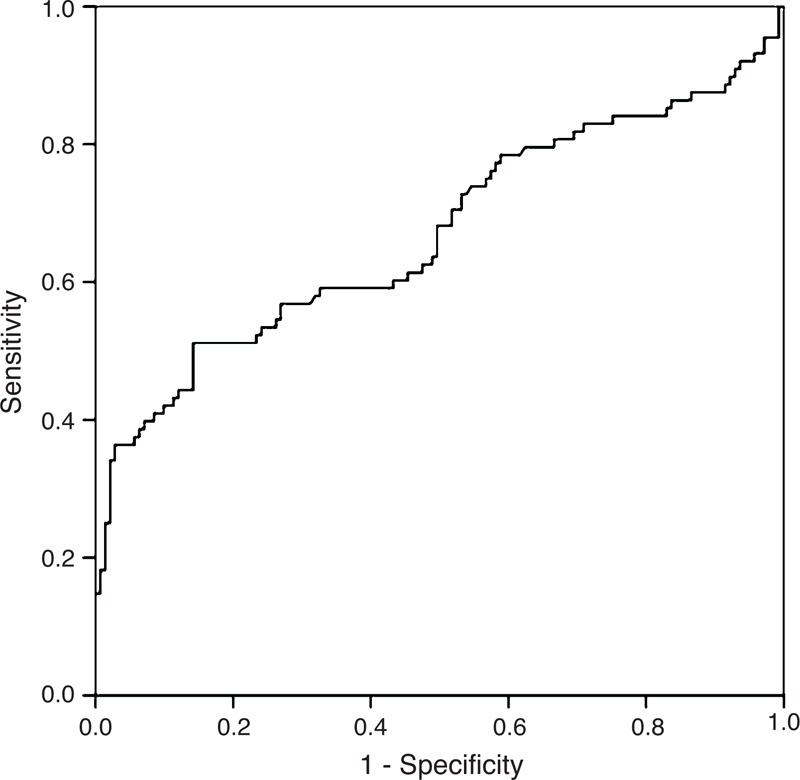
ROC curve obtained with 84 healthy controls and 57 disease-free patients and 88 patients with active disease.

To identify a cutoff value that could distinguish healthy subjects or disease-free patients from patients with active disease, we performed a ROC analysis considering serum CgA levels from the 84 controls and 57 disease-free patients and those from 88 patients with active disease. As shown in Figure 3, the cutoff value of 95 ng/ml provided the best compromise between sensitivity (51.2%) and specificity (87.5%), and was chosen for further analysis. The AUC was 0.678, indicating a good performance of the assay.

### Correlation Between CgA Expression and Serum CgA Levels

CgA positive expression and serum CgA level ≥95 ng/ml were found together in 39 of 88 patients with active disease, the co-positive rate was 44.3% (39/88). Spearman correlation test showed that patients with CgA expression had a significantly high serum CgA levels (*R* = 0.320, *P* = 0.002).

### Relationship Between Serum CgA and Clinicopathologic Factors

Serum CgA levels of patients who classified as NEC + MANEC, were significantly higher than NET patients (106 ng/ml vs. 51 ng/ml, *P* = 0.035). There was a difference in serum CgA levels between patients who were on stage IV and on other stages (*P* = 0.041). Serum CgA levels had no correlation with sex, age, site, functional status, and tumor grading (Table 2).

**TABLE 2:**
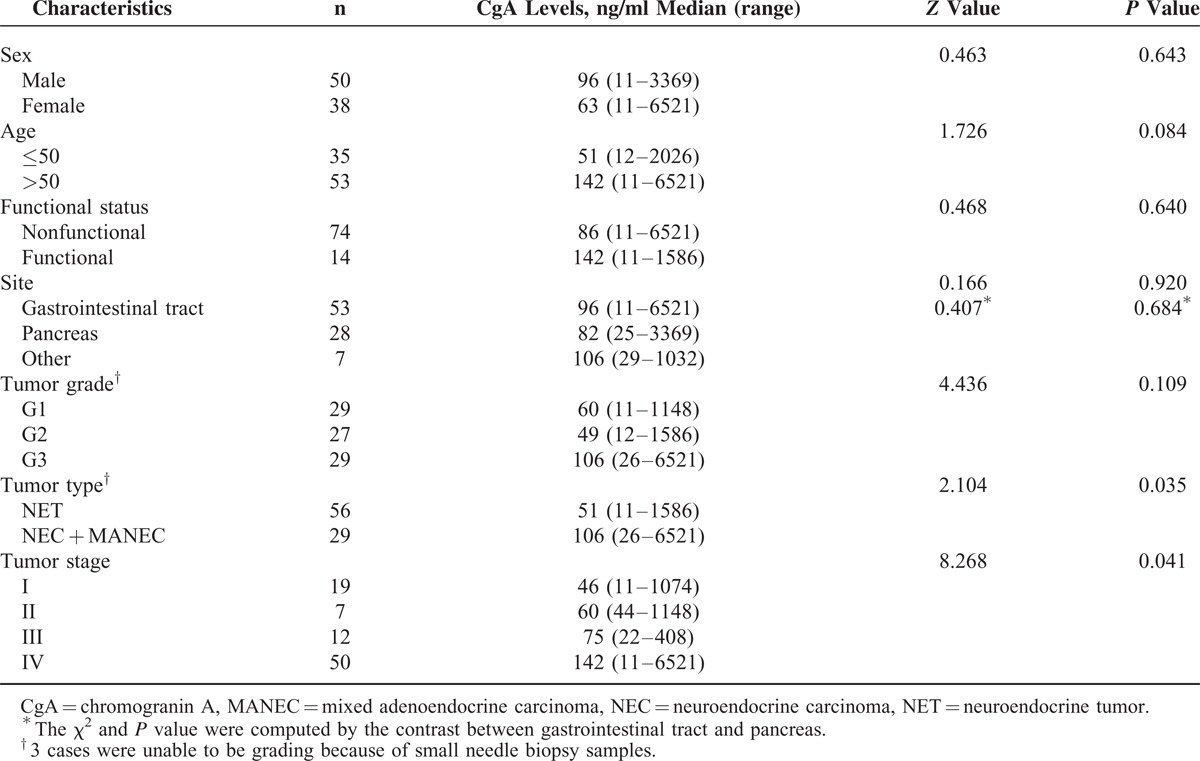
Serum Chromogranin A Levels in Gastroenteropancreatic Neuroendocrine Neoplasm Patients With Active Disease and Correlation With Clinicopathologic Variables (N = 88)

### Changes of CgA in Patients With Active Disease Before and After Treatment

**FIGURE 4:**
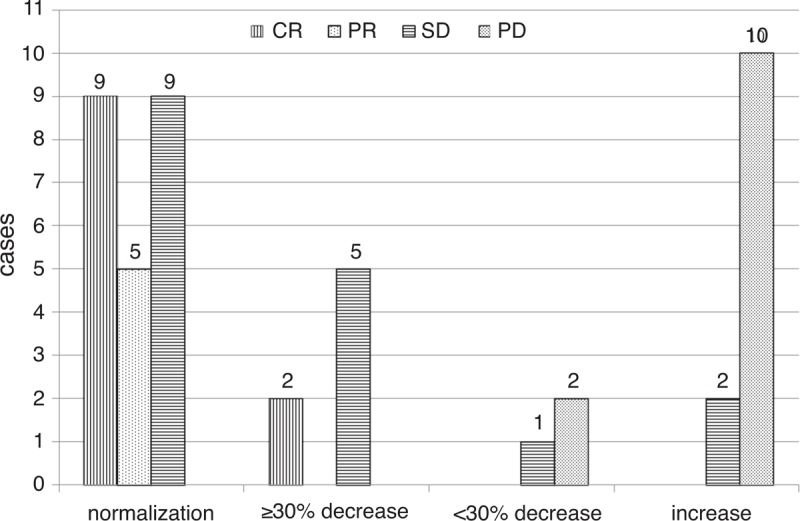
Changes in CgA levels compared with the baseline level (N = 45). CR = complete remission, PD = progressive disease, PR = partial response, SD = stable disease.

Serial CgA levels were performed every 2 to 3 months for 45 patients who received curative surgery or systemic therapy, to evaluate their curative effects. Eleven patients (24.4%) underwent radical surgery had complete remission (CR); five patients (11.1%; two of them treated with somatostatin analog, two treated with sunitinib, and the remainder of patients underwent palliative surgery) had a partial response (PR); other 6 patients administered somatostatin analog therapy, 9 cases received chemotherapy and 2 patients received sunitinib had stable disease (SD; 37.8%); the remainder of 12 patients (26.7%) who received chemotherapy (7 cases), transcatheter hepatic arterial chemoembolization (1 cases), sunitinib therapy (1 cases), and tumor recurrence after surgery (3 cases) had progressive disease (PD). Compared with baseline values, normalization or ≥30% decrease in CgA levels were observed in patients with CR, PR, and SD (30/30, 100%), and <30% decrease or increase in CgA levels were observed in patients with PD (12/15, 80%; Figure 4).

### Survival Analysis

**FIGURE 5:**
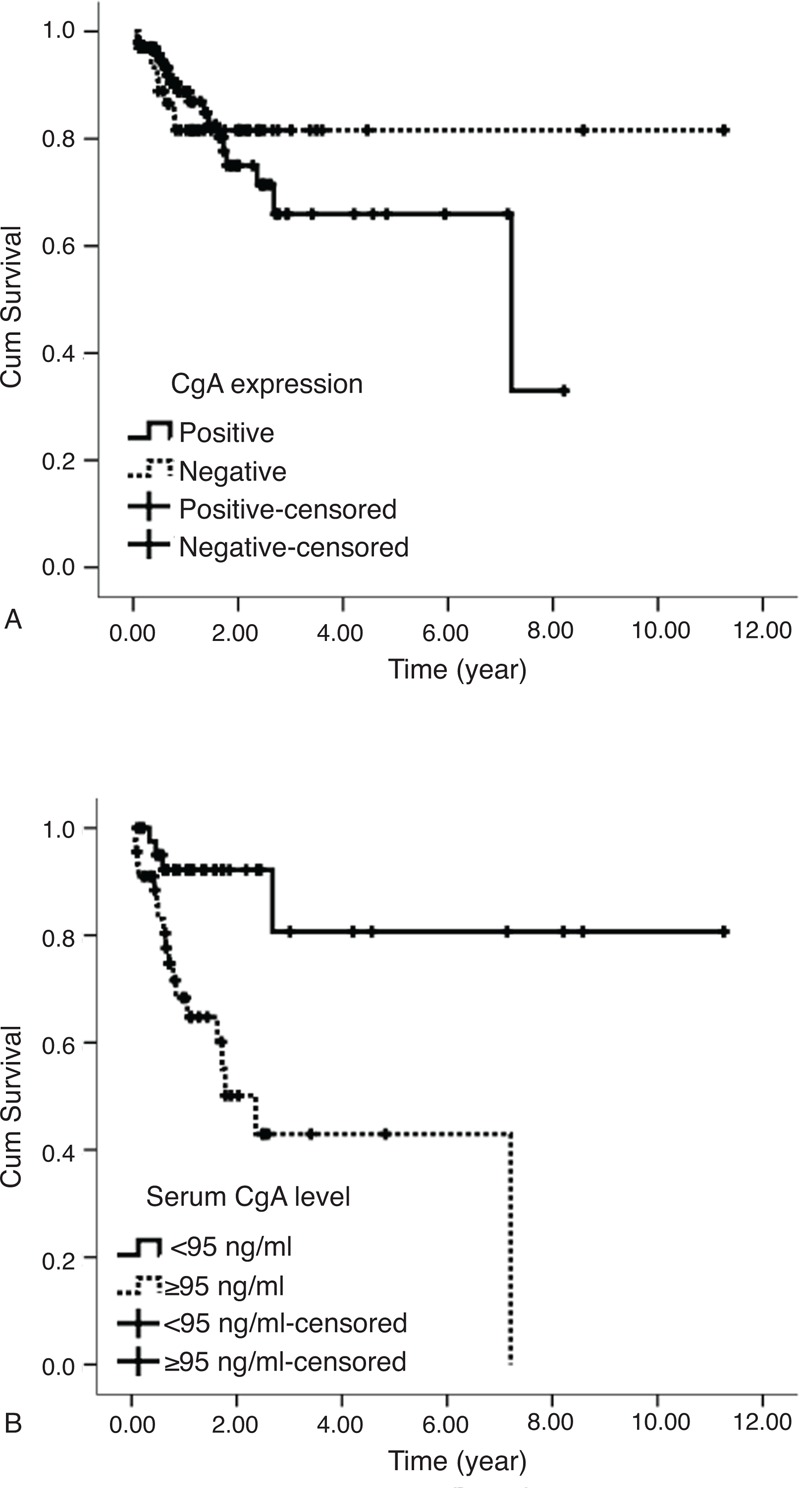
(A) Overall survival by CgA expression and (B) overall survival by serum CgA level. CgA = chromogranin A.

All patients received long-term follow-up with a median duration of 1.13 years (range 0.02–11.26 years). A total of 26 patients died from tumor progress during follow-up (17.9%). Kaplan–Meier survival curves showed that CgA expression was unrelated with prognosis (χ^2^ = 0.316, *P* = 0.574) (Figure 5A). Eighty-eight patients with active disease were divided into serum CgA levels ≥95 ng/ml and serum CgA levels <95 ng/ml groups. The median follow-up time was 1.05 years (range 0.02–11.26 years). As shown in Figure 5B, patients with serum CgA levels ≥95 ng/ml had a significantly shorter survival compared with patients with levels <95 ng/ml (median survival 2.4 years versus not yet reach, χ^2^ = 12.445, *P* < 0.001).

## DISCUSSION

In this study, we have focused on assessing the value of CgA as a marker for diagnosis, monitoring treatment response, and prognosis of GEP-NEN. We found that the overall expression rate of CgA was 69.0%. CgA expression was not associated with patients’ sex, age, functional status, tumor grading, and tumor type. However, we found that CgA expression was probably lower in gastrointestinal neuroendocrine neoplasms than in pancreatic neuroendocrine neoplasms. Especially the expression rate of CgA in rectal NENs was only 37.8% (14/37). Previous studies also have confirmed that CgA expression is usually lacking in rectal NENs because of the specific cell types of this neoplasms.^3^ Additionally, we found that CgA expression was not associated with prognosis. Since CgA is highly expressed and found throughout the diffuse neuroendocrine system in GEP-NEN, it has come to be used as a crucial marker for the pathologic diagnosis of GEP-NEN.^2–4^

CgA not only plays an important role in pathologic diagnosis, but also used as a circulating biomarker. Several studies have demonstrated CgA levels elevated in functional tumors as well as nonfunctional tumors. The sensitivity and specificity rates of CgA for detecting GEP-NEN have been reported to be within the range of 60% to 100%.^5–8^ Chou et al^5^ reported that the sensitivity and specificity of CgA for detecting NEN were 86% and 88%, respectively. Campana et al found that patients with endocrine tumors showed higher levels of CgA than healthy participants, with a sensitivity of 85.3% and a specificity of 95.8%.^8^ In line with previous findings, our study showed that serum CgA level of patients with active disease was significantly higher than of those who were disease-free and healthy subjects. Using a best cutoff value 95 ng/ml, we obtained a sensitivity of 51.2% and a specificity of 87.5%. However, the sensitivity in our study was a little lower than that in other studies. One possible explanation is that CgA expression is usually lacking in rectal NENs, but often high in NENs from midgut origin.^3,17^ In this study, we enrolled 18 rectal NENs out of 88 patients with active disease, but only 4 patients from midgut origin. CgA levels were elevated in only 3 cases of 18 rectal NENs (16.7%), but elevated in 75% (3/4) midgut NENs. Furthermore, a recent study found that serum levels of CgA were not significantly elevated in patients with insulinomas.^18^ In the present study, our findings were very similar to their results which showed only a small part of patients with insulinomas (1/5, 20%) had an increased level of CgA. For these possible reasons, the sensitivity in our study was relatively low. However, our findings demonstrated that CgA was a reliable biomarker for detecting GEP-NEN.

Thirty-nine of 88 patients with active disease were found CgA positive expression and serum CgA levels ≥95 ng/ml at the same time. Spearman correlation test showed that there was a correlation between the CgA IHC expression and high serum CgA levels (*R* = 0.320, *P* = 0.002). To the best of our knowledge, it is the first study confirming the relationship between both of them.

Previous studies have shown that levels of CgA vary with the degree of differentiation and tumor burden. A study by Nolting et al found that GEP-NEN patients affected by liver metastases had significantly higher median CgA values than those without liver metastases (389 ± 38, 103 ng/mL *vs.* 65 ± 181 ng/mL; *P* < 0.0001).^19^ Another study conducted by Walter et al also reported that CgA levels were significantly increased in patients with metastatic disease rather than limited or regional lymph nodes (74% *vs.* 51%; *P* = 0.02).^20^ In agreement with previous findings, our results revealed that CgA levels were increased in patients who were defined as stage IV when compared with those in other stages. In contrast with previous studies, our findings did not support the concept that CgA values were higher in well-differentiated NET than in poorly differentiated tumors.^21^ Interestingly, we found that serum CgA levels of patients with poorly differentiated tumors which were classified as NEC + MANEC, were much higher than well-differentiated NET patients (106 ng/ml *vs.* 51 ng/ml, *P* = 0.035). A possible reason was that 72.4% of NEC + MANEC patients presented with distant metastases, which might result in higher serum CgA levels, while only 48.2% of NET patients presented distant metastases.

CgA has been suggested to be useful for evaluating treatment response to a given therapy such as radical surgery, chemotherapy with fluorouracil + streptozocin + doxorubicin, somatostatin analog, and targeted therapy drug everolimus, for certain subgroup patients.^5,7,22–24,5,7,21,22^ A study assessed CgA levels in 11 patients being treated with somatostatin analog for residual or metastatic GEP-NEN, showed that changes in CgA levels ≥25% predicted SD or a PR to treatment.^5^ For pancreatic NEN patients receiving everolimus therapy, Yao et al reported that a ≥30% reduction in CgA levels within 4 weeks was significantly associated with better median disease progression-free survival compared with those who did not receive therapy (13.3 vs. 7.5 months). In our study, we observed that after different therapies including surgery, chemotherapy, somatostatin analogs therapy, and targeted therapy, normalization or ≥30% decrease in CgA levels suggested CR, PR, and SD, and <30% decrease or increase in CgA levels indicated tumor progression. These findings suggested that serial measurement of CgA might be valuable for assessing therapeutic response in patients with GEP-NEN during follow-up.

Previous studies looking at circulating CgA as a prognostic factor have shown conflicting results. Ardill and Erikkson reported that if CgA levels were greater than 5000 μg/l, prognosis of patients was significantly poorer than those with CgA levels lower than this threshold (*P* < 0.01).^10^ Although increasing CgA levels were revealed as being closely correlated with mortality during follow-up on univariate analysis (*P* = 0.007) by Ahmed et al, they were not identified as an independent predictor of mortality in the multivariate analysis (*P* = 0.923).^11^ In a study of 38 patients with GEP-NEN conducted by Massironi et al showed that baseline CgA levels were not associated with mortality (*P* = 0.655).^12^ In the present study, serum CgA levels ≥95 ng/ml in patients with active diseases were associated with a significantly shorter survival when compared with CgA levels < 95 ng/ml (*P* < 0.001). Although the results about the relationship between circulating CgA level and the survival were inconsistent, our study indicated that patients with serum CgA levels ≥95 ng/ml had a worse prognosis.

In conclusion, CgA is a reliable pathologic and circulating maker for the diagnosis of GEP-NEN. We further confirmed that serial measurement of CgA may be useful for evaluating the efficacy of different types of therapies in patients during follow-up, and serum CgA level ≥95 ng/ml may serve as a predictor of survival.
